# Simultaneous assessment of myocardial scar and coronary artery disease by navigator-gated 3D fat-suppressed delayed-enhancement CMR: comparison with 2D delayed-enhancement CMR, CT and CAG

**DOI:** 10.1186/1532-429X-14-S1-P302

**Published:** 2012-02-01

**Authors:** Yasuo Amano, Tomonari Kiriyama, Masaki Tachi, Yasuhiro Kobayashi, Tetsuro Sekine, Shinichiro Kumita

**Affiliations:** 1 Nippon Medical School, Tokyo, Japan

## Summary

The aim of this study was to assess the feasibility of navigator-gated 3D fat-suppressed delayed-enhancement CMR (DE-CMR) for simultaneous assessment of the myocardial scar and coronary artery disease, by comparing with 2D DE-CMR, CT, and CAG.

## Background

Simultaneous assessment of myocardial scar or perfusion reduction and coronary artery stenosis is currently performed by side-by-side comparison or image fusion using coronary CTA and myocardial perfusion study, but the misregistration and differences in spatial resolution between imaging methods can be problematic. A CMR technique that can visualize both myocardial scar and coronary artery disease during a single examination may resolve this problem.

## Methods

Sixteen patients underwent a navigator-gated 3D fat-suppressed DE-CMR using a 3.0T imager. The spatial resolution of this imaging was 1.5x1.25x3.0-3.4 mm3 before an interpolation. Inversion recovery and spectrally-selective pulses were used to suppress normal myocardial and epicardial fat signals, respectively. The ability of the navigator-gated 3D fat-suppressed DE-CMR to detect myocardial scar was compared with that of 2D DE-CMR. The signal reduction of coronary artery on the 3D DE-CMR was compared with calcified plaques on CT (n = 13) and significant stenosis (> 75%) on CAG (n = 8). The relationship between myocardial scar and coronary arteries was also assessed in six patients with ischemic cardiomyopathies.

## Results

Twenty-five myocardial scars on 2D DE-CMR, 66 coronary calcified lesions on CT, and six coronary stenoses on CAG were investigated in 16 patients. The navigator-gated 3D fat-suppressed DE-CMR detected 25 (92.6%) scars, 43 (65.1%) arterial signal reduction, and three (50%) coronary artery stenoses. When excluding the coronary arteries that were affected by respiratory artifacts, 84.3% of coronary artery diseases were detected by the 3D-CMR. This imaging showed the diffuse stenoses of coronary arteries, comparable to diffuse calcified plaques, in the patients with ischemic cardiomyopathy.

## Conclusions

Navigator-gated 3D fat-suppressed DE-CMR was feasible for simultaneous assessment of the myocardial scar and coronary arteries with calcified plaques.

## Funding

No disclosure for this presentation.

**Figure 1:**
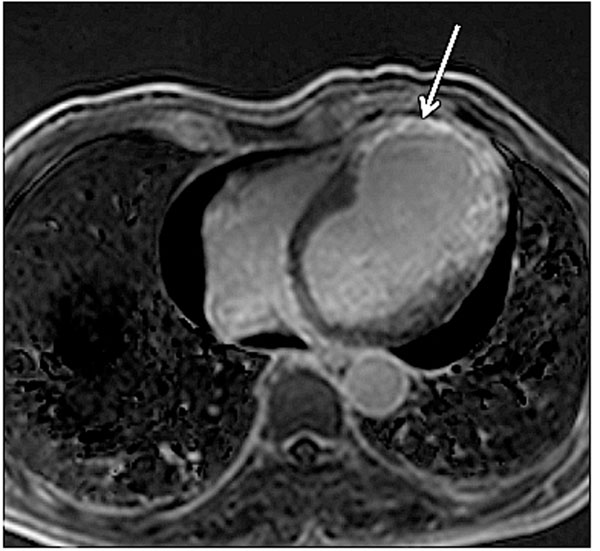
Navigator-gated 3D fat-suppressed DE-CMR shows myocardial scar following infarction.

**Figure 2:**
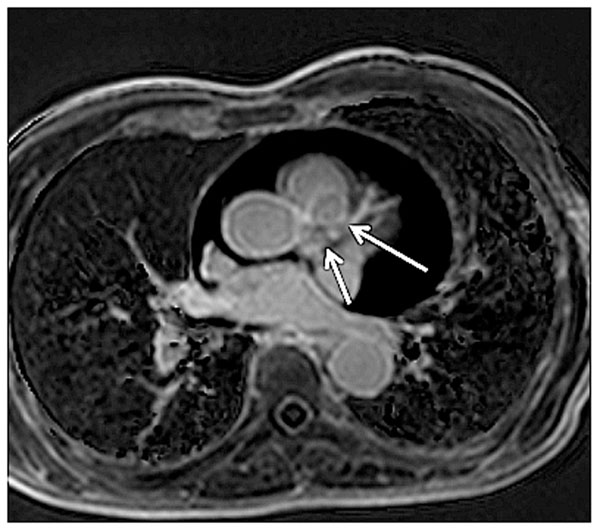
Navigator-gated 3D fat-suppressed DE-CMR shows signal reduction of coronary arteries simultaneously.

